# Chemokine Receptor Antagonists Prevent and Reverse Cofilin-Actin Rod Pathology and Protect Synapses in Cultured Rodent and Human iPSC-Derived Neurons

**DOI:** 10.3390/biomedicines12010093

**Published:** 2024-01-01

**Authors:** Thomas B. Kuhn, Laurie S. Minamide, Lubna H. Tahtamouni, Sydney A. Alderfer, Keifer P. Walsh, Alisa E. Shaw, Omar Yanouri, Henry J. Haigler, Michael R. Ruff, James R. Bamburg

**Affiliations:** 1 Department of Biochemistry and Molecular Biology, Colorado State University, Fort Collins, CO 80523, USA; tom.kuhn@colostate.edu (T.B.K.); laurie.minamide@colostate.edu (L.S.M.); lubna.tahtamouni@colostate.edu (L.H.T.); keiferwalsh@gmail.com (K.P.W.); alisa.shaw@colostate.edu (A.E.S.); 2 Department of Biology and Biotechnology, Faculty of Science, The Hashemite University, Zarqa 13133, Jordan; 3 Department of Chemical and Biological Engineering and School of Biomedical Engineering, Colorado State University, Fort Collins, CO 80523, USA; sydney.alderfer@gmail.com; 4 Molecular, Cellular and Integrative Neuroscience Program, Colorado State University, Fort Collins, CO 80523, USA; omar.yanouri@colostate.edu; 5 Creative Bio-Peptides, Inc., 10319 Glen Road, Suite 100, Potomac, MD 20854, USA; hhaigler@creativebiopeptides.com (H.J.H.); mruff@creativebiopeptides.com (M.R.R.)

**Keywords:** cofilactin rods, chemokine/cytokine receptor antagonists, human iPSC-derived neurons, neurodegenerative proteinopathies, prion-dependent lipid rafts, amyloid-β, IL-6, HIV gp120, peptide-T, DAPTA

## Abstract

Synapse loss is the principal cause of cognitive decline in Alzheimer’s disease (AD) and related disorders (ADRD). Synapse development depends on the intricate dynamics of the neuronal cytoskeleton. Cofilin, the major protein regulating actin dynamics, can be sequestered into cofilactin rods, intra-neurite bundles of cofilin-saturated actin filaments that can disrupt vesicular trafficking and cause synaptic loss. Rods are a brain pathology in human AD and mouse models of AD and ADRD. Eliminating rods is the focus of this paper. One pathway for rod formation is triggered in ~20% of rodent hippocampal neurons by disease-related factors (e.g., soluble oligomers of Amyloid-β (Aβ)) and requires cellular prion protein (PrP^C^), active NADPH oxidase (NOX), and cytokine/chemokine receptors (CCRs). FDA-approved antagonists of CXCR4 and CCR5 inhibit Aβ-induced rods in both rodent and human neurons with effective concentrations for 50% rod reduction (EC_50_) of 1–10 nM. Remarkably, two D-amino acid receptor-active peptides (RAP-103 and RAP-310) inhibit Aβ-induced rods with an EC_50_ of ~1 pM in mouse neurons and ~0.1 pM in human neurons. These peptides are analogs of D-Ala-Peptide T-Amide (DAPTA) and share a pentapeptide sequence (TTNYT) antagonistic to several CCR-dependent responses. RAP-103 does not inhibit neuritogenesis or outgrowth even at 1 µM, >10^6^-fold above its EC_50_. N-terminal methylation, or D-Thr to D-Ser substitution, decreases the rod-inhibiting potency of RAP-103 by 10^3^-fold, suggesting high target specificity. Neither RAP peptide inhibits neuronal rod formation induced by excitotoxic glutamate, but both inhibit rods induced in human neurons by several PrP^C^/NOX pathway activators (Aβ, HIV-gp120 protein, and IL-6). Significantly, RAP-103 completely protects against Aβ-induced loss of mature and developing synapses and, at 0.1 nM, reverses rods in both rodent and human neurons (T_½_ ~ 3 h) even in the continuous presence of Aβ. Thus, this orally available, brain-permeable peptide should be highly effective in reducing rod pathology in multifactorial neurological diseases with mixed proteinopathies acting through PrP^C^/NOX.

## 1. Introduction

Alzheimer’s disease (AD) is the leading cause of dementia, currently affecting 6.7 million Americans age 65 and older [1]. However, more than 50% of people diagnosed with AD have mixed dementia from multiple causes, which includes, among others, cerebrovascular disease (5–10%), Parkinson’s dementia (3.6%), Lewy Body disease (5%), and HIV-associated neurocognitive disorder (HAND) (<1%). The major pathologies of these dementias arising from secondary protein aggregation [2] are characterized as: amyloidopathies, amyloid plaques accumulating from secreted Amyloid-β peptides cleaved from the transmembrane amyloid precursor protein (APP); tauopathies, accumulation of hyperphosphorylated tau into neuropil threads and/or neurofibrillary tangles [3]; and α-synucleinopathies, accumulation of α-synuclein fibrils into Lewy bodies. Each of these pathologies may occur together or in different brain regions and thus have various effects on either or both cognitive and motor function. There are no effective cures for these diseases, and even the recently FDA-approved Lecanamab, an expensive monoclonal antibody infusion to reduce amyloid plaques, has shown adverse effects in some patients. Questions have been raised about both its efficacy and safety [4], as well as the relevance of amyloid plaques as the correct target for the treatment of dementia [5]. Thus, there has been a great deal of attention directed at finding new treatments for these multiple proteinopathies. 

Antagonists of the chemokine receptors CCR5 and CXCR4 have been identified as translational targets to improve recovery after brain injuries [6] as well as cognition in HIV patients, many of whom suffer from HAND [7,8]. The CCR5 antagonist Maraviroc reduced amyloidogenesis, tau pathology, and neurodegeneration in an HIV-infected mouse line that carried human blood leukocytes [9]. In mouse models of AD, subcutaneous administration of the CXCR4 antagonist AMD3100 ameliorated cognitive impairment [10]. Thus, targeting a common disease-related pathology induced by signaling via these CCRs might identify novel therapeutic reagents to treat multiple proteinopathies.

In AD, rod-shaped bundles of cofilin-saturated actin filaments (cofilactin) are a pathological feature found within the hippocampus [11,12]. Because permeabilization of aldehyde-fixed tissue with non-ionic detergents, such as Tween-20 or Triton X-100, inhibits their immunolabeling with cofilin antibodies, rods often go unobserved in aldehyde-fixed tissues [13]. Cofilactin rod pathology has been identified in rodent models of multiple proteinopathies, including AD [14,15], α-synucleinopathy arising from overexpression of alpha-synuclein (α-Syn) [16], intracerebroventricular administration of the HIV envelope gp120 protein linked to HAND [17], and astrocytic overexpression of the neuroactive peptide endothelin-1 [18,19]. In cultured rodent neurons, physiologically relevant amounts (nM or less) of amyloid-β (Aβ), especially the soluble and most synaptotoxic dimer/trimer (Aβd/t), the HIV gp120 protein, and preformed fibrils of α-Syn, induce cofilactin rods [16,20,21,22]. These factors also lead to synaptodendritic loss in vivo [23,24,25,26]. A neuroinflammatory response accompanies the above diseases/disorders, and rods are induced in cultured rodent hippocampal neurons by several proinflammatory cytokines [21]. Rods disrupt vesicular transport and impair synaptic function [27,28,29]. Reducing rod pathology in mouse models of AD eliminates the decline in hippocampal spatial memory [14,15,30] making rod elimination a worthwhile therapeutic target to protect synapses [31,32]. In the present study we, have examined the actions of select chemokine receptor antagonists to block the formation of cofilactin rods induced by activators of the PrP^C^/NOX pathway relevant to Alzheimer’s disease and related disorders (ADRD) in both rodent and iPSC-derived human neuronal cultures. 

Even under optimal conditions for the culture of rodent hippocampal neurons, a small neuronal population (~5%) will form spontaneous rods. However, an additional 20% of neurons will form rods induced by the above-mentioned disease-related factors through a common pathway that includes cellular prion protein (PrP^C^), NADPH oxidase (NOX) [17,21,22], and multiple chemokine/cytokine receptors (CCRs), including CXCR4 and CCR5, which are present on the neurons [22,33,34] as well as on glia [35]. PrP^C^ is a glycosyl-phosphatidylinositol (GPI)-linked protein on the outer leaflet of the plasma membrane that associates with cholesterol and sphingolipid-enriched patches within the membrane (lipid rafts). Disease-associated factors, such as amyloid-β (Aβ) oligomers, especially the synapse disrupting Aβ dimer/trimer (Aβd/t) in AD [20,25,36,37], associate with PrP^C^, a necessary step in the downstream signaling leading to synaptic loss [38,39,40]. Cholesterol binding motifs in many transmembrane proteins, including multiple G-protein coupled receptors (GPCRs; e.g., the metabotropic glutamate receptor mGluR5 and CCRs that bind different tropic forms of the HIV gp120 protein) and transmembrane NOX isoforms, associate with lipid rafts. NOX produces reactive oxygen species (ROS) when cells expressing CCRs are treated with gp120 protein [22,41], and inhibitors of NOX2, a major neuronal isoform involved in adaptive and innate immune responses [42], block rod formation from all of the inducers discussed above. PrP^C^ expression is necessary for cognitive impairment in mouse models of AD [43]. Furthermore, intraperitoneal administration of antibodies directed against PrP^C^ reached effective concentrations in the brain to rescue synapses and cognition in AD mice [44]. Overexpression of PrP^C^ drives rod formation in over 50% of cultured rodent hippocampal neurons even in the absence of external stimuli [21], suggesting rod formation via PrP^C^ signaling might be a driver of cognitive dysfunction.

ROS generated by NOX likely contributes to neurite rod formation and stability through both the dephosphorylation of inactive pSer3-cofilin by activating the cofilin phosphatase slingshot [19,45] and the formation of disulfide-linked cofilin dimers found in rods isolated from neurons that were induced by treatment with sodium azide/2-deoxy-glucose [46,47]. Ectopic expression of the rod reporter, cofilinR21Q-mRFP, has been used in neurons to examine the dynamics of cofilactin structures using fluorescence recovery after photobleaching (FRAP) in both growth cones and intraneurite rods induced by Aβd/t [22,29]. In growth cones, the time to 50% recovery is about 1 min whereas in cofilin-actin rods in neurites, it is about 1 h, strongly suggesting that there are structural differences between cofilactin bundles in these two domains. Reversal of induced rods in cultured rodent neurons has been observed following washout of the inducer or blockage of ROS production by NOX even in the presence of the inducer [21], suggesting that therapeutics preventing rod formation might also be able to reverse preexisting rod pathology.

In this study, we examined the efficacy of rod inhibition and reversal in both rodent neurons and those derived from human-induced pluripotent stem cells (iPSCs) [34] by FDA-approved CXCR4 antagonist AMD3100 (Plerixafor^®^, Sanofi-Aventis, Paris, France) [10,48] and CCR5 antagonist Maraviroc (Selzentry^®^, Pfizer, New York, NY, USA) [49], both of which have beneficial effects on cognitive function in animal models [6,50] We compared these chemokine receptor antagonists to several D-amino acid receptor-active peptides (RAPs), which have also been identified as antagonists of CXCR4 and CCR2/CCR5/CCR8 receptors [51,52]. The peptides are orally stable analogs of Peptide T and DAPTA (D-Ala-Peptide T-Amide) [53,54,55], which were active in the prevention of: (1) lesion-induced cortical atrophy in aged rats [56]; (2) hippocampal microglial and astrocyte activation in a rat model of AD [57]; and (3) synapto-dendritic damage and behavioral delays associated with gp120 treatment in developing rodents [23]. In HIV patients, Peptide T improved brain glucose metabolism [58] and enhanced cognitive performance [7,59,60] Benefits were also shown with Maraviroc [8]. The activity of Peptide T is contained in the C-terminal penta-peptide (TTNYT). We therefore tested five all-D-amino acid pentapeptides and one octapeptide analog for efficacy in reducing Aβd/t-induced rods in both rodent and human neurons. The most active of these peptides was tested at picomolar concentrations for its ability to reverse induced rods in live neurons in the continued presence of rod inducers and to protect against Aβd/t-induced loss of synapses.

## 2. Materials and Methods

### 2.1. Materials

All chemical reagents were obtained from Sigma-Aldrich (St. Louis, MO, USA) unless indicated otherwise. Unless specified elsewhere, tissue culture reagents and immunocytochemistry reagents were from Thermo-Fisher (Waltham, MA, USA). Primary antibodies include: affinity purified rabbit antibody (1 μg/mL, rabbit 1439) made against chick actin depolymerizing factor (ADF) but which is a mammalian pan ADF/cofilin antibody [61]; phospho-Tau mouse monoclonal antibody 12E8 (2 ng/μL) (Elan Pharmaceuticals, Dublin, Ireland); chicken polyclonal IgY to MAP2 (ab92434; 1:1000) (Abcam, Waltham, MA, USA); mouse monoclonal IgG to neurofilament heavy chain (NF-H) (SMI31 801601; 1:600 of 1 mg/mL) (BioLegend, San Diego, CA, USA); goat polyclonal IgG to PSD95 (SC 8575; 1:100 of 0.1 mg/mL) (Santa Cruz Biotechnology, Dallas, TX, USA); rabbit IgG to VGLUT1, a vesicular glutamate transporter (135303; 1:300 of 0.5 mg/mL) (Synaptic Systems GmbH, Goettingen, Germany). Alexa fluor secondary antibodies (Thermo-Fisher; all at 1:500) with various Alexa fluors include: goat anti-rabbit IgG, goat anti-mouse IgG, goat anti-chicken IgY, donkey anti-goat IgG, donkey anti-rabbit IgG, donkey anti-mouse IgG, and donkey anti-chicken IgY.

### 2.2. Isolation of Aβd/t 

The predominantly dimer/trimer fraction of a secreted human amyloid-β (Aβd/t) was derived from a Chinese hamster ovary-cell line (7PA2) [62]. Medium from these cells collected after overnight incubation was concentrated 10-fold on Amicon Ultra 15 mL, 3K cut-off spin filters (Sigma-Aldrich), and the concentrate fractionated on a Superdex-75 Increase (Sigma-Aldrich) gel filtration column equilibrated in 50 mM ammonium acetate buffer [20,24]. The fractions containing Aβd/t were identified by Western blots using an Aβ antibody (6E10; 1:1000) (Covance, Princeton, NJ, USA). Fractions were freeze-dried, resolubilized in 1 mL of sterile water, and freeze-dried a second time for storage. The Aβd/t fraction was reconstituted to 1X (concentration in 7PA2 culture medium, assuming full recovery of the dimer/trimer fraction) in neuronal culture medium prior to addition to neuronal cultures. Quantification of the total Aβd/t pool was determined to be about 1 nM (monomer equivalent) by comparison on immunoblots to a standard curve using synthetic Aβ [20].

### 2.3. Preparation of CCR Antagonists

#### 2.3.1. Preparation of Maraviroc and AMD3100 Stock Solutions

AMD3100 was dissolved in sterile water to make a 1 mM stock, which was further diluted in culture medium for addition to neuronal cultures at the final desired concentration. Maraviroc was dissolved in absolute ethanol to make a 1 mM stock and was then diluted in water or medium such that ethanol was never present in neuronal cultures at greater than 0.1%. Vehicle controls were included in many experiments that showed the absence of an effect of ethanol on rod formation or neurotoxicity. 

#### 2.3.2. Preparation of RAP Peptides 

Peptides 1–6 (see Table 1) were synthesized by ABclonal Technology (Woburn, MA, USA) as trifluoroacetate salts (92% to 98% purity). Method for accurate weighing of fluffy powders is in the Supplemental Material. Peptides 1, 2, 3, and 5 were dissolved in 1 mL of sterile water. The initial stocks were made to 10^−4^ M (higher concentrations may aggregate), and further dilutions to 10^−6^ to 10^−8^ M were made in water within a few minutes for quick freezing (liquid nitrogen) in cryo-vials and storage at −20 °C. Peptides 4 and 6 were dissolved in 100% DMSO (cell freezing grade) to make 5 × 10^−4^ M solutions that were further diluted in sterile water to make 10^−6^ to 10^−8^ M stocks. Dilution of peptides to 100× their final use concentration was made in culture medium (see below) and used at 10 μL/mL medium.

### 2.4. Neuronal Cultures

#### 2.4.1. Rodent Neuron Cultures 

Although some neuronal cultures were prepared from freshly dissected and dissociated E16.5 mouse or E18 rat hippocampi, most cultures were prepared from stocks of dissociated embryonic hippocampal neurons frozen at 10^6^/mL in cryovials at about 1 °C/min in 10% DMSO, 50% HyClone fetal bovine serum (FBS; Thermo-Fisher), and 40% complete homemade Neurobasal (hNB) medium overnight at −80 °C prior to storage in liquid nitrogen. Homemade neurobasal medium (hNB) contains all of the components of commercial Neurobasal medium [63] but is substituted with highly purified L-serine (Bachem AG, Bubendorf, Germany), adjusted to a final concentration of 175 μM L-cysteine, 2.5 mM glucose, and 122 mM NaCl (a final osmolarity of 320 mOsM) [22]. Complete hNB is hNB containing GlutaMAX-1 (R&D Systems, Minneapolis, MN, USA) (25 μL/10 mL), 50 U/mL penicillin, 50 μg/mL streptomycin (Pen/Step mix; Thermo-Fisher), and N21-MAX (R&D Systems) (1 mL/50 mL) [34].

Cultures of dissociated rodent hippocampal neurons were established in serum-free, complete hNB. Frozen neurons were rapidly thawed (37° water bath), immediately diluted at least 1:6 with complete hNB to lower the DMSO concentration to below 2%, and plated in FBS-containing medium (10% HyClone FBS final concentration in complete hNB) onto a German glass surface (Carolina Biological Supply, Burlington, NC, USA) precoated for 60 min at room temperature or overnight at 4 °C with 150 μL per well of 100 μg/mL poly-D-lysine (PDL) in borate buffer (50 mM H_3_BO_3_, 12.5 mM Na_2_B_4_O_7_) in a glass bottom 24-well plate, or with 100 μL per 12 mm round coverslip in wells of a plastic 24-well plate or within a 12 mm hole in a glass bottom 35 mm culture. Each coated surface was washed three times with phosphate-buffered saline (PBS: 140 mM NaCl, 2.7 mM KCl, 8 mM NaH_2_PO_4_, pH 7.2). After incubation of the neurons for 2–3 h (37 °C, 5% CO_2_), medium was exchanged for serum-free complete hNB. Full changes of medium were performed every 2 days in vitro (DIV 2), and treatments were initiated at either DIV 5 or 6 and maintained for 24 to 48 h.

In some short-term cultures used for measuring neurite outgrowth, when the medium was first changed to complete hNB, it contained the neuron-specific fluorescent vital dye NeuO (STEMCELL Technologies, Seattle, WA, USA) [64], which was left on the neurons for 2 h. It takes overnight for this dye to result in brightly labeled neurons, and fluorescence lasts about 48 h [65], but a second 2 h labeling can be conducted on DIV 2. Neuritogenesis and outgrowth could be followed over this time frame. 

For long-term (3-week) rodent hippocampal neuronal cultures, complete hNB for feeding the neurons was conditioned in 10 cm culture dishes containing rodent glia prepared from either rat or mouse cortex [66]. Treatment additives were mixed with harvested glial-conditioned complete hNB before addition to the neuronal cultures.

#### 2.4.2. Human Neuron Cultures

Human neurons were derived from iPSC WTC-11 cells, in which a doxycycline-regulated promoter drives the expression of the Ngn2 transcription factor for glutamatergic neuronal differentiation [67,68]. Following treatment with doxycycline on day 0, neurons require about 55 days in culture to give a strong Aβd/t-induced rod response [34]. Differentiated neurons were plated at 60,000 per well and cultured for 55 days in glial-conditioned complete homemade neurobasal medium (hNB) either on poly-D-lysine/Matrigel (Corning Life Sciences, Tewksbury, MA, USA) coated glass bottom 24-well plates (Cellvis, Mountain View, CA, USA) or on similarly coated 12 mm German glass coverslips placed in plastic 24-well plates. Survival and differentiation of these neurons were >95% [34]. Glial cultures could be used for conditioning medium for about 3 weeks before replacement. Detailed treatments of cultures are provided in Figure Legends.

### 2.5. Expression and Application of Cofilin-mRFP in Rodent Neurons 

E18 rat neurons were infected on DIV 3 with adenoviruses (100 multiplicity of infection (moi)) for expression of cofilin-mRFP (WT) [29]. Adenoviruses were made using the AdEasy system, with viruses tittered using the E2a immunoassay [69,70,71]. At 6 DIV, rods were induced by a change of medium to that containing Aβd/t. Rods that formed after overnight incubation were visualized by live-cell imaging on an incubation stage (Tokai Hit, Shizuoka, Japan) of a fluorescence microscope (BZ-710X, Keyence Corp. of America, Itasca, IL, USA) and were followed at 4 min intervals for ~150 min in the presence and absence of the CCR antagonists, AMD3100 [50 nM] and RAP-103 [0.1 nM].

### 2.6. Immunocytochemistry

#### 2.6.1. Immunolabeling of Cultured Neurons

Cultured neurons were exposed to inhibitors and/or Aβd/t for 20–24 h prior to fixation for 30 min with 4% formaldehyde ± 0.1% glutaraldehyde in cytoskeletal buffer [72], washed 3 times with PBS, and permeabilized with −20 °C methanol (3 min while warming to RT). Methanol permeabilization without the use of non-ionic detergents is critical for optimal observation of rods [13]. After removing the methanol, neurons were washed twice in PBS, twice in Tris Buffered Saline (TBS: 10 mM Tris, pH 7.5, 150 mM NaCl), and incubated for 1 h at RT with blocking buffer (2–5% goat or donkey serum, 1% bovine serum albumin (BSA) in TBS). Primary antibodies (rabbit 1439 anti-cofilin, mouse anti-neurofilament, and chicken anti-MAP2) were diluted in 1% BSA in TBS and incubated overnight on cells at 4 °C. After 3–5 washes with TBS, secondary fluorescent-labeled antibodies (goat anti-rabbit Alexa 488 or 647, goat anti-mouse Alexa 564, or goat anti-chicken Alexa 488 or 564) ± DAPI (1:500 of 1 mg/mL) were added for 1 h at RT in 1% BSA/TBS. After 3–5 TBS washes, coverslips were mounted onto glass slides with Prolong Diamond Antifade ± DAPI (Thermo-Fisher) and air dried overnight in the dark. 

To visualize spines and synapses, mouse and human neuronal cultures were fixed and permeabilized as described above and blocked with 5% donkey serum. Primary antibodies: chicken polyclonal IgY to MAP2, goat polyclonal IgG to PSD95, and rabbit IgG to VGLUT1. Secondary antibodies used: donkey anti-goat Alexa 647, donkey anti-rabbit Alexa 568, and donkey anti-chicken Alexa 488. 

#### 2.6.2. Immunolabeling Cofilactin Rods in the Human Alzheimer’s Hippocampus

A short postmortem interval (<2 h) of formaldehyde-fixed hippocampal brain tissue was obtained from the University of Kentucky Alzheimer’s Disease Center Rapid Autopsy Program. A gross examination of the brain to confirm Alzheimer’s pathology was performed by a neuropathologist, and the sample used here was from a subject with confirmed Alzheimer’s disease. Following cryoprotection at 4 °C in 10% sucrose in PBS for 1–2 days and then in 20% sucrose in PBS overnight, tissue was frozen in O.C.T. Compound (Ted Pella, Inc., Redding, CA, USA) and 30 μm frozen sections prepared on a cryostat and adhered to Superfrost Plus slides (Avantor VWR, Radnor, PA, USA). Sections were thawed for 15 min at RT and permeabilized with 80% methanol/20% PBS for 90 s, followed by 0.05% Triton X-100 in PBS for 90 s, a suboptimal method but one that allows immunolabeling with multiple antibodies, including those for cofilin in rods [13]. After washing with PBS and blocking 1 h at room temperature in 5% goat serum and 1% BSA in TBS, sections were incubated overnight at 4 °C with primary antibodies (rabbit 1439 anti-cofilin at 1 ng/μL, 12E8 mouse anti-pTau at 2 ng/μL) diluted in 1% BSA in TBS. Washing and treatment with secondary antibodies were as described above for cultured neurons. After washing off secondary antibodies, sections were treated with 70% ethanol for 5 min and 0.1% Sudan Black B in 70% ethanol for 12–15 min to reduce autofluorescence [73]. Sections were rinsed briefly twice with 70% ethanol, followed by a rinse with TBS, before applying 25 μL of Prolong Antifade Gold (Thermo-Fisher) and an ethanol-cleaned coverslip.

### 2.7. Image Acquisition and Analysis

#### 2.7.1. Image Acquisition

- Images for rod quantification were acquired on a Keyence BZ-710X microscope using a 20× objective and filter cubes at respective fluorescence wavelengths for DAPI and Alexa dyes. Generally, an array of 7 × 7 fields (approximately 12 mm^2^) was acquired for each coverslip or well of a glass bottom plate using autofocus on the cofilin channel, followed by stitching using Keyence Analyzer software (BZ-H3 AE) into a single multi-fluorescence (overlay) image. Images were captured at standard sensitivity (2 × 2 binning).
- Live imaging of rodent hippocampal neurons expressing cofilin-mRFP or NeuO was performed on the BZ-710X Keyence microscope with either 20× or 40× objective with cultures maintained at 37 °C on a Tokai Hit stage incubation system with CO_2_ set at 5%. Time-lapse capture mode was used for some imaging. NeuO imaging used a filter cube with 470/40 nm excitation, 495 nm dichroic, and 620/60 nm emission [65].
- Images of the human AD brain section and of rods in fixed rodent and human neuronal cultures for synapse analysis were obtained on an Olympus IX83 microscope with either 60 × 1.42 NA or 100 × 1.4 NA objective, equipped with a Yokagawa spinning disc illumination system, lasers at 405, 488, 568, and 647 nm, and an Andor iXon camera, all integrated by Intelligent Imaging Innovations (3I, Denver, CO, USA) operated by Slidebook software (version 2023.3). Three color overlays of MAP2, PSD95, and VGLUT1 immunolabeled neurons were captured for synapse analysis, whereas two color overlays were captured for cofilin and p-tau immunolabeled human AD hippocampal sections.

#### 2.7.2. Rod Quantification

Stitched images of fixed neurons immunolabeled for cofilin and nuclei (DAPI), as well as either NF-H or MAP2, were exported as tiff files to Metamorph (version 7.8, Molecular Devices, San Jose, CA, USA) or ImageJ (Version 1.53e; Java 1.8.0_172; ImageJ.org). In some cases, macro functions were applied to set local thresholds and shape/size segmentation for cofilin rods. In low-density rodent neuronal cultures, rod-like inclusions positive for cofilin were manually counted per stitched array, together with the number of neuronal cell bodies, by an analyzer blind to the treatments. These are reported as rods per neuron. Rod-like staining found at the end of a process was not included in our quantification because these often arise from collapsed growth cones, which contain cofilin-actin bundles [74]. Because growth cones are infrequent in cultures of Day 55 human neurons [34], scoring rods in these cultures did not require this correction. In high-density rodent neuronal cultures or human neurons, either rods per neuron (estimated from DAPI-stained nuclei) or rods normalized to the neurite area (either MAP2 or NF-H immunolabeling) were used. Data points on bar graphs represent results from individual cultures. Combining data from separate experiments required normalizing each data set to the average of the treatment, giving the highest response set as 100% [34].

#### 2.7.3. Neurite Length Measurements

Neurite lengths were determined from either NeuO images of living neurons or fixed neurons with neurites immunolabeled for NF-H. An ImageJ plug-in for morphometric analysis (https://blog.bham.ac.uk/intellimic/g-landini-software/ (accessed on 15 December 2021)) was applied, which removes cell soma from a DAPI image and condenses neurites to a single pixel width such that the total neurite area equals the length of neurites in pixels (Figure S1).

#### 2.7.4. Synapse Quantification 

Synapses in rodent neuron cultures and developing synapses in human neurons were quantified as PSD95 immunolabeled puncta that contact VGLUT1 immunolabeled puncta in deconvolved images. PSD95, a postsynaptic marker, accumulates in spines along MAP2-labeled dendrites in both 21 DIV rodents and Day 55 human neurons. VGLUT1 is a marker of glutamate neurotransmitter vesicles, which accumulate at presynaptic sites in axons. Synapses in rodent neurons were normalized to 20 μm segments of secondary dendrites selected from >40 fields taken at random from multiple coverslips, and treatments were blind to the microscopist. Developing synapses in human neurons were normalized to total dendritic density (MAP2 immunolabel) across entire fields [34]. 

### 2.8. Statistics

Statistical analyses included one-way ANOVA with Tukey’s or Dunette’s posthoc-analysis, non-parametric Kruskal–Wallis between groups comparison, and Student’s unpaired parametric two-tailed *t*-test, all performed using GraphPad Prism (Dotmatics, Boston, MA, USA) to determine significant differences of the means of each condition compared to untreated (control) or to treatments giving the maximum rod response, and across multiple samples or between two treatments, with a *p* < 0.05 being taken as statistically significant. Significant differences are shown on plots as: * *p* < 0.05; ** *p* < 0.01; *** *p* < 0.001; **** *p* < 0.0001; ns = not significantly different.

## 3. Results

### 3.1. Rod Immunolabeling and Morphology Are Identical in Rodent and Human Neurons

**Figure 1:**
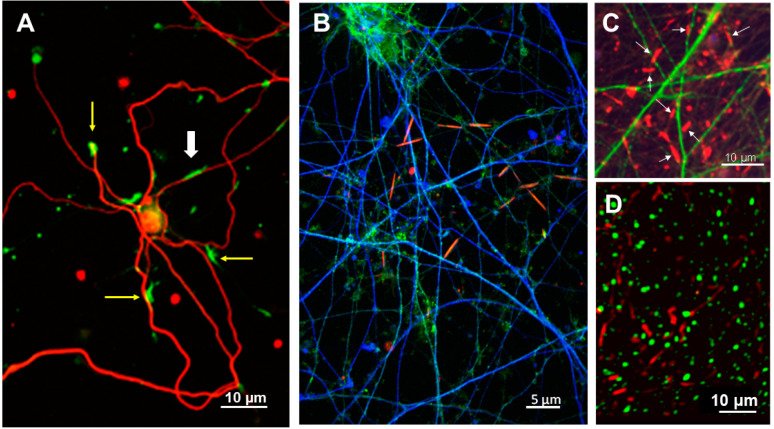
Comparison of cofilactin rod morphology in cultured rodent and human neurons and the human AD brain. (**A**) A 6 day in vitro (DIV) rodent hippocampal neuron treated overnight with Aβd/t showing NF-H immunolabeled neurites (red) and cofilin (green). Image acquired with a 20× air objective. One rod (white arrow) is differentiated from cofilin immunolabeled growth cones (yellow arrows) by its morphology and location. (**B**) Projection image of a confocal stack (60× objective) of Day 55 human iPSC-derived neurons treated overnight with Aβd/t showing cofilin-containing rods (red), MAP2 immunolabeled dendrites (green), and NF-H immunolabeled axons (blue). (**C**) Cofilin immunolabel (red) in cultures of human neurons also immunolabeled for MAP2 (green). Rods (white arrows) disrupt the cytoskeleton in the neurites in which they form, eliminating most microtubules to which MAP2 binds [11,34]. (**D**) Cofilin immunolabeled rods (green) in a hippocampal section from the brain of a confirmed AD subject in a longitudinal AD study. The section is co-immunolabeled for *p*-tau (red), showing the non-overlapping neuropil thread pathology. Thicker (30 μm) tissue images have rods and neuropil threads that are not parallel within the focal plane, and after creating a projection image and processing by deconvolution, both rods and threads have a decreased elongated morphology compared to images of the cultured neurons, in which rods fully reside within a few confocal planes.

Neuronal cofilactin rods are linear bundles of cofilin-saturated F-actin, almost always within neurites. Their morphology is dependent on how they are imaged (Figure 1A–D). When imaged at low magnification (10 to 40× air objectives, using a 2 × 2 binning mode), the rods appear thicker (Figure 1C) than in projection images of confocal stacks taken with 60–100× oil objectives (Figure 1B). Nevertheless, rods in cultured rodent neurons (Figure 1A) and cultured human neurons (Figure 1B,C) are morphologically identical when fixed, immunolabeled, and viewed under identical conditions. They are also very similar in morphology to rods within 30 µm frozen sections of cofilin immunolabeled human AD hippocampus in that they do not overlap with *p*-tau neuropil thread pathology (Figure 1D). 

### 3.2. Inhibition of Rod Formation by Cytokine/Chemokine Receptor Antagonists

#### 3.2.1. AMD3100 and Maraviroc

**Figure 2:**
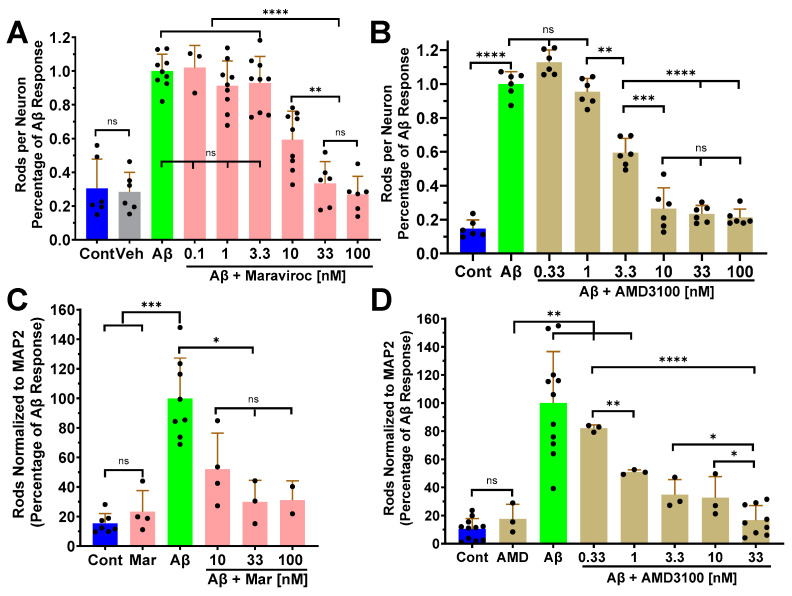
The formation of cofilactin rods induced by Aβd/t is inhibited in a dose-dependent manner by chemokine antagonists. Dose-response determinations were performed in cultured E16.5 mouse hippocampal neurons at 6 DIV ((**A**,**B**); *n* = 3) and in Day 55 human iPSC-derived neurons ((**C**,**D**); *n* = 2) either by Maraviroc (**A**,**C**) or AMD3100 (**B**,**D**). Maraviroc is dissolved in ethanol before aqueous dilution (Veh is the ethanol control; 0.1%). AMD3100 (EC_50_ = 2–3 nM) is ~3 fold more potent than Maraviroc (EC_50_ = 8–9 nM) in inhibiting Aβd/t-induced rods in both mouse and human neurons. Semi-log plots of these data are in (Figure S2). Error bars are the standard deviation (S.D.). * *p* < 0.05; ** *p* < 0.01; *** *p* < 0.001; **** *p* < 0.0001; ns = not significantly different.

Inhibition of CCR5 and CXCR4 receptor function with FDA-approved CCR antagonists Maraviroc (CCR5) and AMD3100 (CXCR4) at >30 nM fully or partially inhibited Aβd/t- and gp120- induced rod formation in rodent and human neurons [22,34]. Here we compared the rod inhibitory potencies of these CCR antagonists from <1 nM to 100 nM on Aβd/t-induced rods in both 6 DIV mouse hippocampal neurons and Day 55 human iPSC-derived glutamatergic neurons (Figure 2). Maraviroc had an approximate EC_50_ of 8–9 nM in both mouse and human neurons, estimated from semi-log plots of the data (Figure S2A,C). AMD3100 was about 3 fold more active in both mouse and human neurons with an EC_50_ of 2–3 nM (Figure S2B,D).

#### 3.2.2. Comparative Activities of All D-Amino Acid Receptor Active Peptides (RAPs)

After quantifying the nM dose-response for inhibition of cofilin-actin rod formation by specific chemokine receptor antagonists, we proceeded to evaluate the rod-inhibiting activities of six peptides (Table 1) that are stable analogs of clinically used DAPTA. The octapeptide RAP-310 (Peptide 5) is an all-D-amino acid version of DAPTA. RAP-103 (Peptide 1) is the pentapeptide sequence contained within the C-terminal region of RAP-310. The four other peptides are modifications of RAP-103, ranging from simple changes, such as the addition of an N-terminal methyl group (Peptide 6) or the substitution of serine for threonine at the N-terminus (Peptide 3), to more extensive D-amino acid substitutions in Peptides 2 and 4.

**Figure 3:**
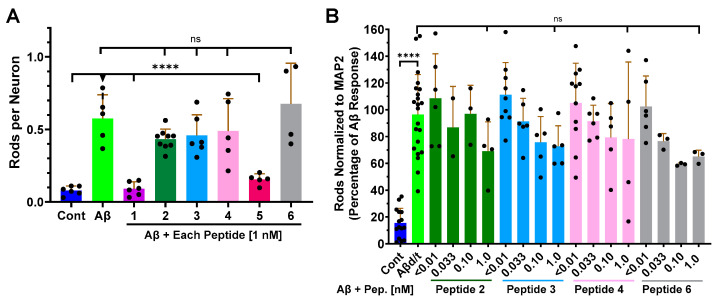
Inhibition of Aβd/t-induced rod formation in mouse hippocampal and human neurons by RAP peptides. (**A**) Rod formation in E16.5 mouse hippocampal neurons (6 DIV) untreated or treated 24 h with Aβd/t in the absence or presence of 1 nM of each RAP peptide (*n* = 2). Significant rod inhibitory activity was observed only for Peptides 1 and 5 (RAP-103 and RAP-310). (**B**) Limited dose-response curves for the 4 less active peptides in Day 55 human neurons A slight trend toward inhibition with increasing peptide concentration did not reach significance (*p* < 0.05) or 50% inhibition at 1 nM (*n* = 2). Error bars are S.D. **** *p* < 0.0001; ns = not significantly different.

Preliminary tests in rodent neurons showed RAP-310 was more active in inhibiting Aβd/t-induced rods than either of the FDA-approved antagonists, so we screened each of the peptides at 1 nM for inhibition of Aβd/t-induced rod formation in mouse hippocampal neurons (Figure 3A). Only RAP-103 and RAP-310 demonstrated significant rod-inhibiting activity at this single concentration. We then performed limited dose-response curves in Day 55 human neurons with the 4 peptides that did not give a rod-inhibiting response in rodent neurons (Figure 3B). They all showed a slight increase in rod inhibition at higher concentrations, but none reached an EC_50_ or a significant level of reduction at 1 nM. 

#### 3.2.3. Dose-Dependent Inhibition of Rod Formation by RAP-103 and RAP-310 

**Figure 4:**
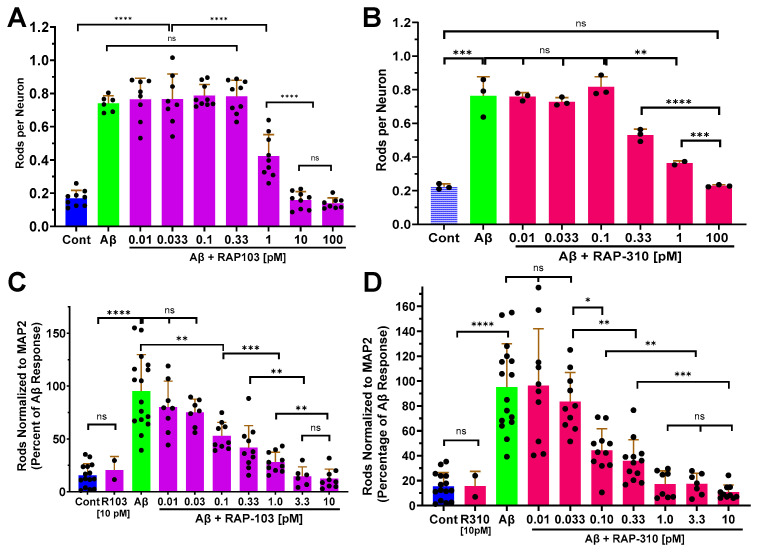
RAP-103 and RAP-310 inhibited the formation of Aβd/t-induced rods in a dose-dependent manner in both rodent and human neurons. Rod quantification in DIV 6 mouse hippocampal neurons (**A**,**B**) or Day 55 human neurons (**C**,**D**) treated 24 h with Aβd/t along with RAP-103 (**A**,**C**) or RAP-310 (**B**,**D**) at concentrations shown. Both RAP peptides had nearly identical rod-inhibiting activity and were about 10-fold more effective in blocking rod formation in human neurons than in mouse neurons (*n* = 3 for (**A**–**D**) and 1 for (**B**)). The EC_50_ values for inhibition of Aβd/t-induced rods were estimated from semi-log plots of these data (Figure S3A–D) to be ~1 pM in mouse hippocampal neurons and ~0.1 pM in human neurons. Error bars are S.D. * *p* < 0.05; ** *p* < 0.01; *** *p* < 0.001; **** *p* < 0.0001; ns = not significantly different.

The potency of RAP-103 and RAP-310 to block Aβd/t-induced rod formation in both rodent hippocampal neurons and human neurons was then tested using the same culture and treatment protocols used for the inactive peptides, but over a broader range of peptide concentrations. For both RAP-103 and RAP-310, rods per neuron were scored in rodent neurons over a 10^5^-fold range [0.01–100 pM], and rods normalized to the MAP2 area were scored in human neurons over a 10^4^-fold range [0.01–10 pM] (Figure 4). 

### 3.3. Studies on RAP Peptide Inhibition of Rods by Other Inducers

**Figure 5:**
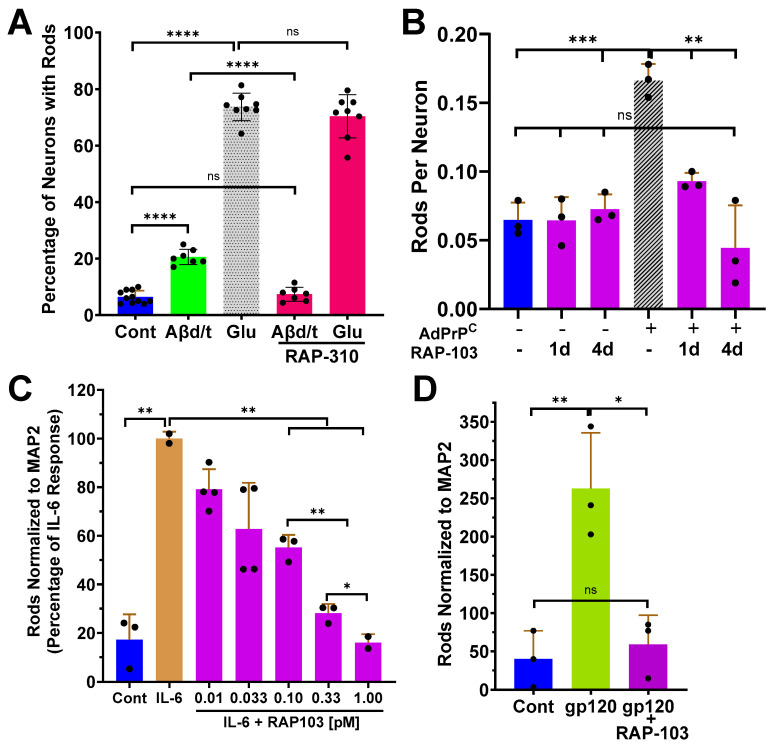
Effects of RAPs on rods induced by other initiators. (**A**) Treatment of 6 DIV rat hippocampal neurons with glutamate [150 μM] for 1 h induced rods in >70% of neurons, whereas overnight treatment with Aβd/t induced rods in a subpopulation of <25%. RAP-310 [100 pM] added with the rod inducer completely inhibited Aβd/t-induced rods but not rods induced by glutamate (*n* = 3). (**B**) Rod formation induced by adenoviral-mediated overexpression of PrP^C^ in rat hippocampal neurons infected on DIV 2 with 100 moi Ad-PrP^C^ and treated with RAP-103 [50 pM] at the same time (4 day) or on DIV 5 (1 day), fixed and immunolabeled on DIV 6, was prevented (4 day) or reversed (1 day) by RAP-103 treatment (*n* = 1 but examined in more depth in Figure 6). (**C**) Rod formation induced in human neurons treated on Day 54 with IL-6 [100 ng/mL] and fixed on Day 55 is inhibited in a dose-dependent manner by RAP-103 with an EC_50_ estimated as 0.1 pM from a semi-log plot of these data (Figure S3E) (*n* = 2). (**D**) Rods induced in human neurons treated on Day 54 with 500 pM dual tropic gp120_MN_ and fixed on Day 55 are inhibited from forming by RAP-103 [10 pM] (*n* = 1). Error bars are S.D. * *p* < 0.05; ** *p* < 0.01; *** *p* < 0.001; **** *p* < 0.0001; ns = not significantly different.

To further examine if the RAP peptides function directly on CCRs, we explored their effects on other rod initiators. To determine if rod inhibition by the RAP-peptides could be acting intracellularly by directly targeting cofilin-actin interactions, we examined the ability of RAP-310 to inhibit cofilactin rods induced by excitotoxic concentrations of glutamate via AMPAR, previously shown to be blocked with an AMPAR antagonist [29]. Although 100 pM RAP-310 fully inhibited Aβd/t-induced rods, it did not inhibit rod formation induced by glutamate (Figure 5A), which utilizes a pathway independent of PrP^C^/NOX [21]. 

To determine if RAP-103 inhibited PrP^C^-induced rod formation, we demonstrated that at 50 pM, RAP-103 blocked (4-day treatment) and reversed (added for last day only) rod formation in DIV 6 rodent neurons induced by 4 days of adenoviral-mediated overexpression of PrP^C^ (Figure 5B). To determine if the active RAPs inhibit rod formation induced by other ligands with well-characterized receptors, we induced rods in Day 55 human neurons with the cytokine interleukin 6 (IL-6) and with the dual tropic form of HIV gp120. RAP-103 inhibited IL-6-induced rod formation with an EC_50_ of ~0.1 pM (Figure 5C and Figure S3E), identical to its EC_50_ for inhibiting Aβd/t-induced rods. Rod formation induced by the dual tropic gp120_MN_ [500 pM] was completely inhibited by 10 pM RAP-103 (Figure 5D).

### 3.4. Reversal of Aβd/t-Induced Rods by RAP-103 and Kinetics of Reversal

**Figure 6:**
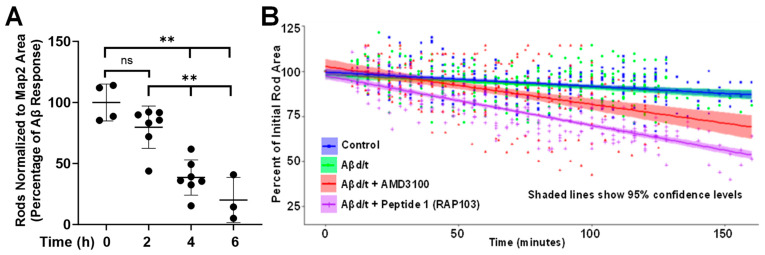
Kinetics of reversal of Aβd/t-induced rods by 100 pM RAP-103 in the continuous presence of the rod inducer. (**A**) On Day 54, human neurons were treated with Aβd/t for 24 h. At 6, 4, and 2 h before fixation, RAP-103 was added to 100 pM. Rods were quantified from multiple coverslips per time point, normalized to the MAP2 area, and the rod response from untreated cultures was subtracted. The time to 50% decrease of Aβd/t-induced rods is ~3 h (*n* = 2). Error bars are S.D. ** *p* < 0.01; ns = not significantly different. (**B**) E18 rat neurons were infected on DIV 3 with adenovirus to express cofilin-mRFP, and on DIV 6, they were left untreated (control) or treated overnight with Aβd/t. Fluorescent rods were identified, and their areas were determined in images collected at 4 min intervals in untreated control cultures (spontaneous rods, blue line) and those to which either AMD3100 [50 nM] (red line) or RAP-103 [0.1 nM] (purple line) was added at time 0. Shaded areas show a 95% confidence level around the average slope determined from multiple rods in each treatment (*n* = 3). Some rods in Aβd/t-treated cultures (green line that overlaps the blue line) did not respond to either AMD3100 or RAP-103 and had a slope identical to that for spontaneous rods in control cultures.

Previous studies indicated reversal of rods induced in rodent hippocampal neurons by TNFα occurred following washout of the inducer [21]. Rods induced by Aβd/t were reversed in human neurons in the continued presence of Aβd/t after 24 h of treatment with AMD3100 (30 nM) [34]. Here the time-course for RAP-103 reversal of rods induced by 24 h of Aβd/t treatment was determined by rod quantification in Day 55 human neurons kept in the presence of Aβd/t to which RAP-103 was added 6, 4, and 2 h before fixing (Figure 6A). The time to 50% rod reversal in human neurons is ~3 h. 

Direct observation of rod disappearance was performed in E18 rat neurons infected on DIV 3 with adenovirus for expressing cofilin-mRFP. When the levels of cofilin-mRFP fluorescence became just detectable by fluorescence microscopy, usually on DIV 6, neurons were incubated overnight with Aβd/t or remained untreated (control). On DIV 7, rods were identified by live-cell microscopy, and fields containing rods were imaged every 4 min over a 2.5 h period without treatment (control) or following the addition of AMD3100 [50 nM] or RAP-103 [0.1 nM] (Figure 6B). There was no significant difference in the decline in rod size between control neurons (which contain only spontaneous rods) and those induced by Aβd/t and in its continued presence. This decline is due to photobleaching since rods outside the field of illumination maintain their fluorescence and size. Both RAP-103 and AMD3100 reversed rods induced by and in the continued presence of Aβd/t, but RAP-103 did so at a 2-fold faster rate even though it was used at a 500-fold lower concentration. The extrapolated time to 50% decrease in rod size is ~3 h, which correlates with the slow resumption of vesicular transport in neurites observed in a previous study using the cofilin R21Q-mRFP rod-reporter [29]. The observed slope of the spontaneous rods can be used to correct for photobleaching, which results in an adjusted time of 50% loss of rod area to about 200 min for RAP-103. 

### 3.5. RAP-103 Inhibits Aβd/t-Induced Synapse Loss in Cultures of Mouse and Human Neurons

**Figure 7:**
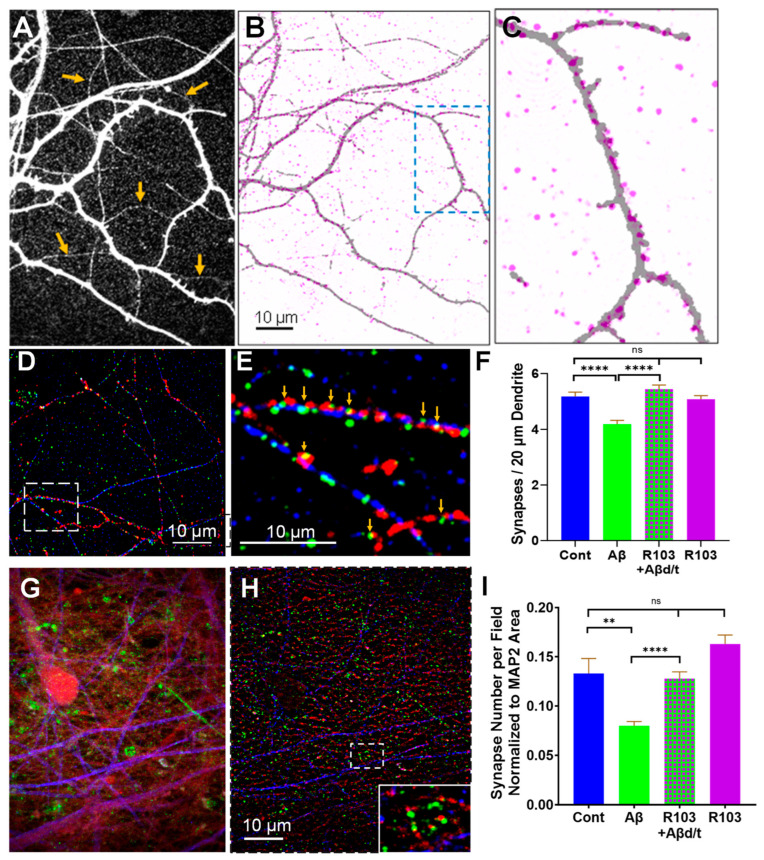
RAP-103 protects against Aβd/t-induced loss of mature and developing synapses in cultured mouse and human neurons. Mouse E16.5 hippocampal neurons were cultured in a glial-conditioned medium for 21 days. Cultures were either untreated (control) or treated with Aβd/t ± RAP-103 [0.1 nM] on DIV 18, fixed on DIV 21, and immunolabeled for MAP2, PSD95, and VGLUT1. Projection images of 2 μm confocal stacks (0.1 μm/plane) were obtained, and deconvolved images of PSD95 and VGLUT channels (colorized green and red, respectively) were used for synapse analysis. To demonstrate that the PSD95 puncta observed in deconvolved images overlay dendrites, a b/w image of the MAP2 channel (**A**) was overlayed with the PSD95 puncta from an inverted image (**B**) with a magnified region of the boxed area shown in (**C**). Both strongly and weakly labeled dendrites (orange arrows in (**A**)) show almost all PSD95 puncta (pink) align with dendrites, even though some MAP2-positive dendrites are too faint to show up on the deconvolved overlays. (**C**) Most spines in this enlarged image are MAP2 positive and immunolabel for PSD95. (**D**–**F**) Deconvolved confocal projection image (**D**) showing MAP2 (pseudocolor blue), VGLUT1 (pseudocolor red), and postsynaptic PSD95 (pseudocolor green). (**E**) Enlarged boxed area from (**D**) showing synapses where VGLUT1/PSD95 directly abut or overlap (orange arrows). (**F**) Synapses were quantified per 20 μm segment of 5 secondary dendrites per field from ~50 fields from each of 4 coverslips (205–230 segments) per treatment. Error bars are SEM. **** *p* < 0.0001; ns = not significantly different. Other than treatment with Aβd/t alone, no other treatment is significantly different from the control. (**G**–**I**) Day 50–51 human neurons were untreated (control) or treated for 4–5 days with Aβd/t ± RAP-103 [0.1 nM] or RAP-103 alone, before fixation and immunolabeling. (**G**) Non-deconvolved confocal projection image of Day 55 human neurons immunolabeled and pseudocolored for MAP2 (blue), PSD95 (green), and VGLUT1 (red). (**H**) Deconvolved image in (**G**) with magnified boxed region as inset. (**I**) Quantification of PSD95/VGLUT1 contacts over entire fields, each normalized to the area of MAP2 immunolabel to correct for dendrite density between fields and summarized for each treatment. Treatments with the number of fields analyzed (*n* = coverslips for each treatment): Control: 118 (*n* = 5); Aβd/t: 143 (*n* = 6); Aβd/t + RAP-103: 167 (*n* = 7); RAP-103: 72 (*n* = 3). Error bars are SEM. ** *p* < 0.01; **** *p* < 0.001; ns = not significantly different. Scale bars in (**B**,**H**) also apply to (**A**,**G**), respectively.

Rodent hippocampal neurons 21 DIV and Day 55 human neurons grown with astrocytes develop active glutamatergic synapses [25,67,75]. Human iPSC-derived neurons mature in the absence of astrocytes and form dendritic spines that make axonal contacts where presynaptic vesicles containing VGLUT1 accumulate, but these do not become functional synapses in the absence of glia [34]. Cultures of both neuronal types were grown to maturity in 24-well glass bottom plates in glial-conditioned complete hNB to assess the ability of RAP-103 to protect against the loss of synapses observed previously in the apical dendrites of rat hippocampal pyramidal neurons in slice cultures treated for 5–15 days with Aβd/t [25]. MAP2 immunolabeled dendrites with associated PSD95 immunolabeled postsynaptic densities are shown in deconvolved images of 21 DIV mouse neurons (Figure 7A–C).

PSD95 puncta are in spines along dendrites [76], but many dendrites are weakly immunolabeled for MAP2 and disappear on the overlay images. PSD95 puncta have an associated MAP2 immunolabel from within the dendritic spine. Since rod formation is maximal between 12–24 h after treatment of cultured hippocampal neurons with Aβd/t [20], we treated mouse neurons for only 3 days ± RAP-103 [0.1 nM] to focus on the shorter-term effects of rods. We quantified synapses within 5 × 20 μm segments of secondary dendrites per field from 4 coverslips for each treatment (205 to 230 segments/treatment) (Figure 7D–F). Developing synapses were also quantified in Day 55 human neurons, but the treatment time was increased to 4–5 days with Aβd/t ± RAP-103 [0.1 nM]. Untreated cultures and those treated with RAP-103 alone were also included. Developing synapses (PSD95/VGLUT contacts) were quantified and normalized for dendrite density across the entire field using the MAP2 immunolabeled area (Figure 7G–I). The very significant decline in developing synapses induced by Aβd/t in Day 55 human neurons was completely prevented by 0.1 nM RAP-103, which by itself induced no alteration in developing synapses. Thus, in both rodent and human neurons, RAP-103 protects against Aβd/t-induced synaptic loss at the same concentration at which it fully inhibits cofilactin rod formation.

### 3.6. RAP-103 Has No Negative Effects on Neuritogenesis and Early Neurite Outgrowth

Before examining the effects of RAP-103 on neuritogenesis, outgrowth, and survival, we first determined that Aβd/t induced a significant rod response in young (DIV 2–3) E18 rat hippocampal neurons, about doubling the percentage of neurons with rods (Figure S4A). However, this response is only about 25–30% of what occurs in DIV 6–7 cultures, in which the percentage of neurons with Aβd/t-induced rods increases 4–5 fold over controls [20,21,22]. We utilized live imaging of NeuO-labeled neurons between DIV 2 and 4 to compare neurite outgrowth between cultures treated with RAP-103, Aβd/t, or both together, but no significant differences were found (Figure S4B–D). We then compared neuron survival between untreated cultures and those treated for 2 days (DIV 2–4) with 1 μM of RAP-103, RAP-310, AMD3100, Maraviroc, or a vehicle control (Figure S4E). No significant difference in the neuronal density of 45 ± 10 neurons/5 mm^2^ was observed between treatments. Neurite length per neuron was also not significantly different between treatments (Figure S4F). However, there was a significant difference in the percentage of neurons showing additional neurite outgrowth between DIV 4 and 6 for treatments with 1 μM RAP-310 or AMD3100 (Figure S4G). It is doubtful, however, that these are biologically significant since total outgrowth did not differ. It is worth noting that for these studies, the RAPs were used at ~10^6^-fold above their rod inhibiting EC_50_, whereas Maraviroc and AMD3100 were used at ~10^3^-fold above theirs. 

## 4. Discussion

Here we show that cofilactin rod formation induced in cultured rodent and human neurons by agents implicated in dementias from multiple initiators involves a complex multi-component receptor system utilizing PrP^c^/NOX signaling. The intracellular rods disrupt the normal neurite cytoarchitecture [11,27,34] and synaptic function distal to their location [28]. Loss of synapses strongly correlates with cognitive decline, and many different mechanisms that could mediate synapse loss have been proposed [77], to which we add cofilactin rod formation. Rod formation induced by treatment with Aβd/t is not fully inhibited by either CXCR4 antagonist AMD3100 or CCR5 antagonist Maraviroc when used at 10 nM, but AMD3100 is about 3–5-fold more potent than Maraviroc. However, rod induction by either Aβd/t or the proinflammatory cytokine IL-6 is fully inhibited by 1 pM. RAP-103 or RAP-310, oral forms of DAPTA, an early inhibitor of the chemokine receptors CCR5 and CXCR4 [55]. Furthermore, RAP-103 protects against Aβd/t-induced synapse loss in cultures of both rodent and human neurons, and its ability to protect developing synapses in the absence of glia indicates a neuron-autonomous effect (Figure 7). Additionally, both RAP-103 and RAP-310 have a similar EC_50_ for rod inhibition, demonstrating that the activity of RAP-310 is contained within the C-terminal five amino acids of the octapeptide, and thus they are likely to have the same target. Both RAP-103 and RAP-310 block Aβd/t-induced rod formation with a 10-fold lower EC_50_ in human neurons than in rodent neurons, and both are 10^3–^10^4^-fold more active rod inhibitors than AMD3100 or Maraviroc. RAP-103 also has 10^3^-greater activity than Peptides 3 and 6, each of which has only a slight N-terminal structural modification, suggesting a highly specific interaction between RAP-103 and its target(s). We focused mostly on RAP-103, the smaller of the two peptides, because of its likely better brain entry [78] and lower cost to manufacture. These peptides, or their predecessor, DAPTA, target chemokine/cytokine-dependent processes in other cell types with the same potency at which they inhibit rod formation [51,52,79]. 

Induction of rods by specific HIV gp120 protein agonists of CXCR4 and CCR5, and inhibition by AMD3100 [22] and here by RAP-103, is relevant to earlier results of the clinical peptide DAPTA to block gp120 synapto-dendritic damage and improve cognition and brain imaging in early HIV trials [7,54]. Presently, we can only speculate on how RAP-103 inhibits rods induced by PrP^C^ overexpression as well as structurally unrelated molecules. However, its potency in rod inhibition from these diverse inducers suggests that RAP-103 targets signaling via PrP^C^-rich domains, through which CCR signaling is also dependent, and thus provides a broader spectrum for synapse protection than more limited chemokine receptor-specific antagonists. 

Rods are induced through the PrP^C^/NOX/CCR pathway by structurally diverse molecules, including TNFα, IL-1β, IL-6, HIV gp120 proteins, and endothelin-1, which engage innate immune system receptors. Notably, the cellular prion protein PrP^C^ itself functions as a damage pattern recognition receptor on neurons and microglia for synaptotoxic oligomers of soluble Aβ [39], tau [80], and α-Syn [81]. It is noteworthy that all receptors of rod-inducing ligands, such as the proinflammatory cytokine TNFα, accumulate within lipid raft domains enriched in cholesterol and sphingolipids [82]. IL-6-induced rods are inhibited from forming in human neurons by RAP-103 at the same EC_50_ (0.1 pM) as inhibition of rods induced by gp120 or Aβd/t. Furthermore there are no additive effects on rod formation when mixtures of rod inducers are used [21], suggesting a common mechanism for rod inhibition by RAP-103. What could this mechanism entail? 

In rodent hippocampal neurons expressing ectopic PrP^C^, rod formation reached a level that was not further increased by the addition of Aβd/t or TNFα [21], suggesting that the ligands function to locally increase PrP^C^ density to a critical level. Additionally, ligands for innate immune system receptors fail to induce rods in neurons that are null for either PrP^C^ or p47^PHOX^, a cytoplasmic protein subunit of NOX required for activity of NOX 1–3 isoforms [83]. Furthermore, RAP-103 and RAP-310 inhibit rod formation from all PrP^C^/NOX-dependent rod-inducers, including overexpressed PrP^C^. Thus, the signaling platform provided through PrP^C^ is the key element in rod formation. However, in human glutamatergic neurons derived from an iPSC line, it takes several weeks for the development of an Aβd/t-induced rod response [34]. Although this response is enhanced in 2–3-week cultures by ectopic expression of PrP^C^, a strong rod response to Aβd/t that is independent of ectopic PrP^C^ did not develop until 4–5 weeks but then doubled again by 8 weeks. The development of the late increased rod response is accompanied by a doubling of both CXCR4 and PrP^C^ protein levels, although other proteins implicated in Aβ-induced synaptic dysfunction, such as the GPCR mGluR5 [38] or PirB [84,85], might prove to be limiting factors for rod formation at earlier times. 

CCR activation starts with its dimerization, which is dependent on its overall receptor density [86]. Once dimerization begins, large arrays of active receptors collect within cholesterol-rich membrane lipid rafts. The transmembrane domains of CXCR4 and CCR5 contain cholesterol-binding (CRAC/CARC) domains [87,88] but also depend upon membrane sphingolipids enriched within PrP^C^-lipid rafts. Depletion of sphingomyelin altered the ability of the natural CXCR4 agonist CXCL12 to mediate directed cell migration [89], whereas depletion of cholesterol led to the loss of CXCR4 clustering in lipid rafts and its inability to promote HIV-uptake [90]. Major and minor ligand-binding pockets are formed by different transmembrane domains of CXCR4, in which antagonists of CXCR4 activation, such as AMD3100, can bind in either pocket or overlap between these but result in the inability of the receptors to dimerize, thus blocking the large array assembly [86]. Activation of the rod-inducing signaling pathway could involve two principal avenues: (1) CCR dimerization and hence initiation by an external ligand, such as what occurs upon treatment with HIV gp120 protein, followed by coalescence of lipid raft domains and further consolidation of the signaling pathway; or (2) coalescence of lipid raft domains, experimentally induced by PrP^C^ overexpression or other ligand-mediated aggregation, which will subsequently increase CCR densities sufficiently to facilitate dimerization/activation in the absence of their specific ligands. In either case, antagonists of the CCRs, such as AMD3100 and Maraviroc, prevent their dimerization/activation at concentrations in the nM range, which is similar to their K_D_ for receptor binding [91] and for their ability to inhibit rod formation (Figure 2). Based upon our results and those of others, we presume rod formation is a stochastic process in which the major components need to coalesce to provide the appropriate environment in which a rod assembles and is stabilized. This process requires: (1) adequate levels of active (dephospho) cofilin; (2) F-actin composed primarily of ADP-actin subunits, which presumably have been fragmented into shorter lengths with the assistance of AIP1 [46,92]; and (3) reactive oxygen species, presumably either for sulfhydryl oxidation leading to cofilin dimers or oxidation of actin, which can alter actin assembly and cofilin-actin interactions [21,47,93]. Levels of locally active cofilin are regulated in part through dephosphorylation of a reserve phospho-cofilin pool as well as the local release of cofilin from the cytoplasmic face of the membrane, where it remains inactive while bound to PI(4,5)P_2_, but from which it can be released upon activation of phospholipase C (PLC) [94,95,96]. PLC activation can be mediated through many pathways, including GPCRs [97] and the src family protein tyrosine kinase fyn, which is activated by Aβ-binding to PrP^C^ [98,99,100]. PrP^C^ is now the favored physiological receptor targeted by Aβ oligomers [40,44,101,102,103]. We suggest rod signaling is disrupted by the prevention of raft recruitment of CXCR4/CCR5 by inhibitors of their dimerization/oligomerization, such as Miraviroc and AMD3100, which work at nM concentrations. However, we suggest that the RAP peptides inhibit rod formation by targeting PrP^C^-lipid raft enlargement and/or NOX recruitment that is mediated by binding of Aβd/t and other rod inducers. We also suggest that this enlargement is necessary to reach a critical level of ROS production and cofilactin for rod assembly. Removal or inactivation of other components within this raft complex also reduces or prevents rod formation. 

Because of the complex regulation of cofilin activity [104], there are also alternative targets that could modulate rod formation downstream of PrP^C^/NOX, and these could have both rod-dependent and rod-independent effects on synaptic function. For instance, cofilin-induced F-actin depolymerization in vivo requires the major neuronal adenylyl cyclase-associated protein (CAP1) [105], which enhances the release of cofilin-actin from filament “pointed” ends while facilitating the exchange of ATP for ADP on the released actin monomer and priming it for reassembly [106,107]. The functional interdependence of CAP1 and cofilin has recently been demonstrated in modulating dendritic spine morphology [108], so signaling that phosphorylates CAP1 and reduces its binding to cofilactin [109,110] might also be a mechanism to stabilize cofilactin and enhance rod formation. 

Rods sequester the vast majority of cofilin within neurites in which they form (Figure 1) and thus may be the driving force for the enhanced “actinification” that has been observed in cultured neurons following activation of the glutamate-sensitive NMDA receptors and in the mouse brain following photothrombic stroke [111]. Indeed, cofilactin rods are characteristic of ischemic brain injury [112,113], and reduction of active cofilin by upregulating LIM kinase is protective of synapses [112,114]. Thus, cofilactin rods induced by different pathways appear to be a major factor altering synaptic function. Although RAP-103 does not reduce glutamate-induced rods (Figure 5A), it is a potent and specific inhibitor for rods induced by ADRD-associated factors that signal through the PrP^C^/NOX pathway.

## 5. Conclusions

Inhibitors of multiple CCRs potently block synapse loss in rodent hippocampal and human iPSC-derived mature neurons caused by Aβd/t. Synapse loss is preceded by the formation in up to 20% of neurons of cofilin-actin rods induced by activators of the PrP^C^/NOX/chemokine receptor pathway that include disease-related factors associated with multiple proteinopathies. The CCR antagonist RAP-103 offers effective synapse protection by inhibiting and reversing cytoskeletal changes induced via PrP^C^/NOX signaling. Furthermore, because mixed proteinopathies may have additive or synergistic contributions to neurodegeneration, with patients exhibiting more severe and rapid disease progression, RAP-103 may offer improved treatment of multifactorial neurological diseases with synapse loss. It is effective at >10^3^-fold lower concentrations than the FDA-approved CCR antagonists, Maraviroc and AMD3100, which target single receptors, and has a broad window (>10^6^-fold) between the lowest effective concentration for complete rod inhibition and the highest concentration tested, which showed no apparent neurotoxicity based upon outgrowth and morphology. RAP-103 can reverse rod pathology induced by PrP^C^ overexpression as well as in the presence of rod inducers such as proinflammatory cytokines and Aβd/t. The oral availability of RAP-103, its pM dosage for full rod reversal, and its safety and efficacy based on clinical studies of its predecessor, DAPTA, make it a promising candidate for development as a therapeutic for AD and related cognitive disorders.

## Figures and Tables

**Table 1:** RAP Peptides Tested for Inhibiting Aβd/t-induced Rods and Synapse Loss.

RAP Peptide	Peptide Number	D-amino Acid Sequence (Residues Identical to RAP-103)	Rodent Neuron EC_50_ [pM]	Human Neuron EC_50_ [pM]
RAP-103	1	TTNYT	1.5	0.1
	2	SSTYR	~1000	~1000
	3	STNYT	~1500	~1500
	4	ETWYS	~500	~500
RAP-310	5	ASTTTNYT	1.8	0.1
	6	N-methyl-TTNYT	>5000	>1000

## Data Availability

All requests for primary data and experimental reagents should be addressed to: james.bamburg@colostate.edu.

## Supplementary Materials

The following supporting information can be downloaded at: https://www.mdpi.com/article/10.3390/biomedicines12010093/s1. Figure S1: Measuring neurite lengths Figure S2: Log dose-response curves for inhibition by Maraviroc and AMD3100 of cofilactin rods induced by Aβd/t in mouse and human neurons. Figure S3: Log dose-response curves for inhibition by RAP-103 and RAP-310 of cofilactin rods induced by Aβd/t in mouse and human neurons. Figure S4: Neurite outgrowth and morphology in rodent neurons exposed to Aβd/t ± RAPs of CCR antagonists at sub nM to μM concentrations.

## Abbreviations

Aβ	amyloid-β peptide derived from the Amyloid Precursor Protein (APP)
Aβd/t	dimer/trimer-containing fractions of Aβ secreted from cells with AD mutations in APP
AD	Alzheimer’s Disease
ADRD	Alzheimer Disease and Related Disorders
ADF	actin depolymerizing factor
AIP1	actin interacting protein-1
α-Syn	alpha-synuclein
AMPA	α-amino-3-hydroxy-5-methyl-4-isoxazolepropionic acid
AMPAR	an AMPA-specific glutamate receptor mediating rapid synaptic depolarization
APP	amyloid precursor protein
BSA	bovine serum albumin
CAP1	(adenylyl) cyclase-associated protein-1
CCD	charged couple device
CCR	chemokine/cytokine receptors including CXCR4 and CCR5
Cofilactin	cofilin-saturated F-actin (1:1)
CRAC/CARC	protein cholesterol-binding domain that functions in either orientation
DAG	diacylglycerol
DAPI	4′,6-diamidino-2-phenylindole (DNA stain)
DAPTA	D-Ala-Peptide T-amide
DIV	Days in vitro
DMSO	dimethylsulfoxide
F-actin	Filamentous actin
FBS	fetal bovine serum
FRAP	fluorescence recovery after photobleaching
GPCR	G-protein coupled receptor
GM1	ganglioside GM_1_
gp120	HIV envelope glycoprotein fragment derived from gp160
GPI	glycosylphosphatidylinositol
HAND	HIV-Associated Neurocognitive Disorder
HIV	human immunodeficiency virus
hNB	homemade Neurobasal medium
IL-6	interleukin 6
IP_3_	inositol 1,4,5-triphosphate
iPSC	induced pluripotent stem cell
MAP2	microtubule-associated protein 2
moi	multiplicity of infection
NF-H	neurofilament heavy chain
NOX	NADPH Oxidase
PBS	phosphate buffered saline
PDL	poly-D-lysine
PLC	phospholipase C
PrP^C^	cellular prion protein
PSD95	post-synaptic density protein 95
RAP	receptor active peptide
Rods	cofilin-saturated F-actin bundles
ROS	reactive oxygen species
S.D.	standard deviation
SEM	standard error of the mean
TBS	Tris-buffered saline
VGLUT1	vesicular glutamate transporter, a presynaptic marker in glutamatergic neurons
