# Supplementary material for: Chemokine Receptor Antagonists Prevent and Reverse Cofilin-Actin Rod Pathology and Protect Synapses in Cultured Rodent and Human iPSC-Derived Neurons
Source: Biomedicines. 2024 Jan 1;12(1):93. doi: 10.3390/biomedicines12010093 (PMC10813319; doi:10.3390/biomedicines12010093)
Supplement: Supplementary file 1 [file biomedicines-12-00093-s001.zip › biomedicines-2752595-supplementary.pdf]

Supplementary Material

Chemokine Receptor Antagonists Prevent and Reverse Cofilin-actin Rod Pathology and Protect Synapses in Cultured Rodent and Human iPSC-derived Neurons

Thomas B. Kuhn, Laurie S. Minamide, Lubna H. Tahtamouni, Sydney A. Alderfer, Keifer P. Walsh, Alisa E. Shaw, Omar Yanouri, Henry Haigler, Michael Ruff, James R. Bamburg

Methods

Weighing out peptides: Static build-up causes fluffy powders such as dried peptides to fly off the spatula and onto other surfaces, especially in a dry climate. To get an accurate weight requires extreme care. An antistatic spray (StaticGuard) was used on a Kimwipe to wipe down the outside of the ultra-analytical balance to neutralize static charge. A grounding strap connected to grounded fixture was worn on the wrist of the ungloved hand of the person holding the metal micro-spatula. The outside of the peptide containing vial was also wiped with antistatic sprayed tissue before opening the vial. A 2 ml plastic weigh boat, also wiped on its underside with antistatic sprayed tissue, was set on the microanalytical balance, the balance reset to zero and a 5-10 mg quantity (accurate to 0.01 mg) of peptide was weighed. Once the weight was recorded, the balance was locked in position and the peptide solubilized in a few hundred microliters of sterile water or DMSO, depending on its solubility. Peptides 4 and 6 required DMSO, the others were water soluble. The weigh boat was then put in a sterile petri dish and transferred to a biological safety cabinet for sterile dilutions of peptide stocks.

Supplementary Figures

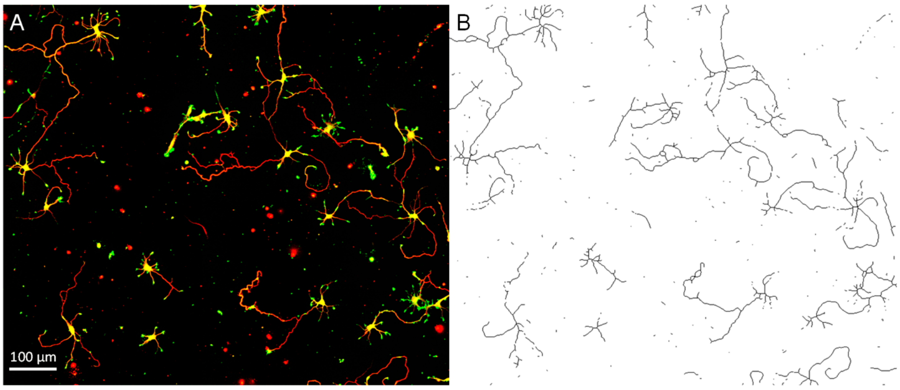

Figure S1. Measuring neurite lengths. (**A**) Overlay images of E18 rat neurons 3 DIV immunolabeled for cofilin (green) and NF-H (red) for which DAPI labeled nuclei were also imaged. For the ImageJ plug-in that reduces neurites to single pixel width, the DAPI image was used as the area for removal of cell bodies (labeled as Nuclear_) and the NF-H channel was used for the neurites (labeled as Neuronal_). (**B**) Output of the ImageJ plug-in (tracings) in which the area provided is equal to neurite length in pixels.

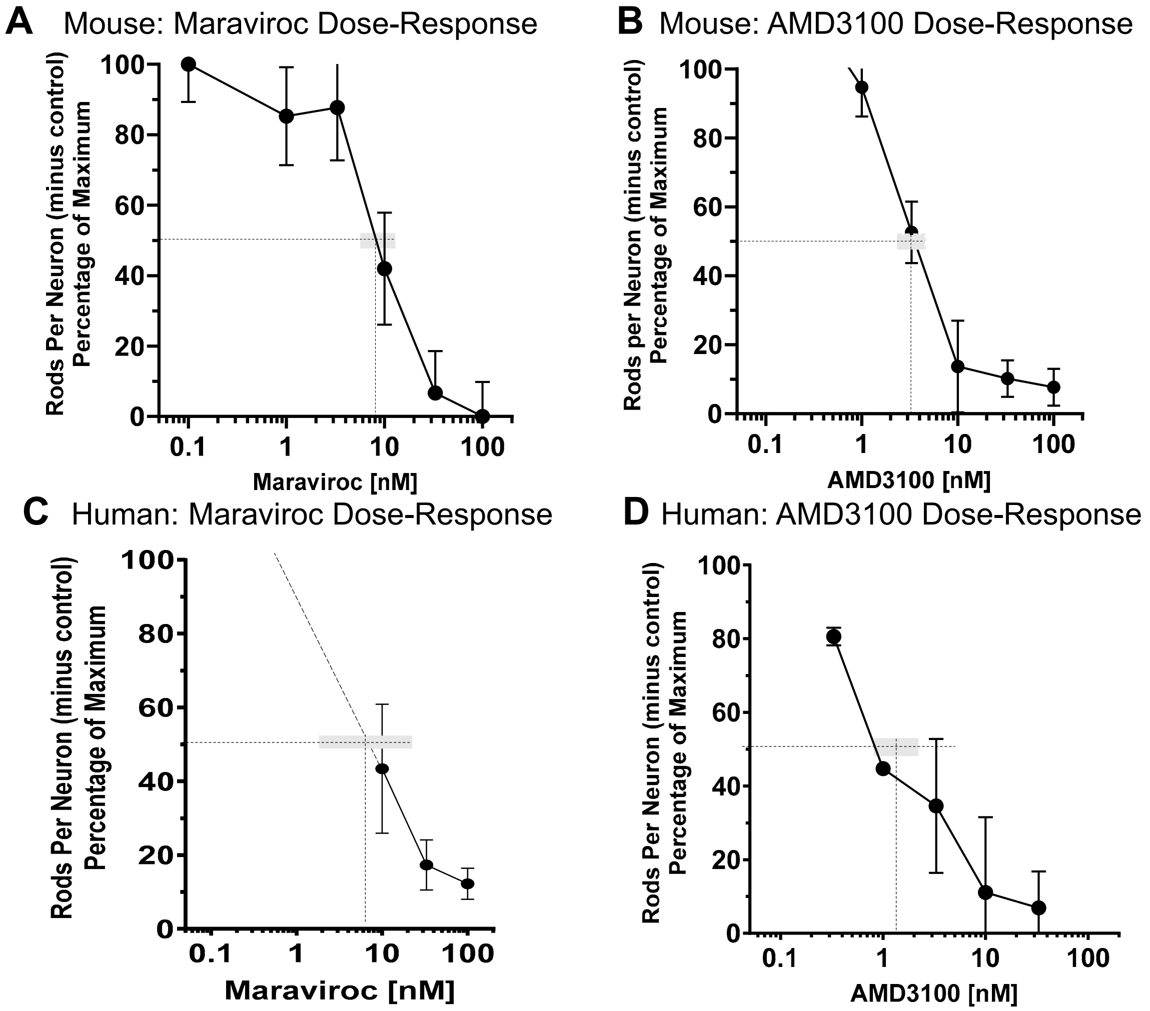

Figure S2. Log dose-response for inhibition of Aβd/t-induced formation of cofilactin rods by Maraviroc and AMD3100 in mouse and human neurons. Data from Figure 2 are replotted with a log dose scale for the drugs after subtracting the control rod-response from the response at different concentrations of drug and renormalizing the rod values to the highest response set as 100%. The ranges for the likely EC_{50} values calculated from the S.D. of points on each plot are shown by the gray bar across the horizontal dashed line at 50%.

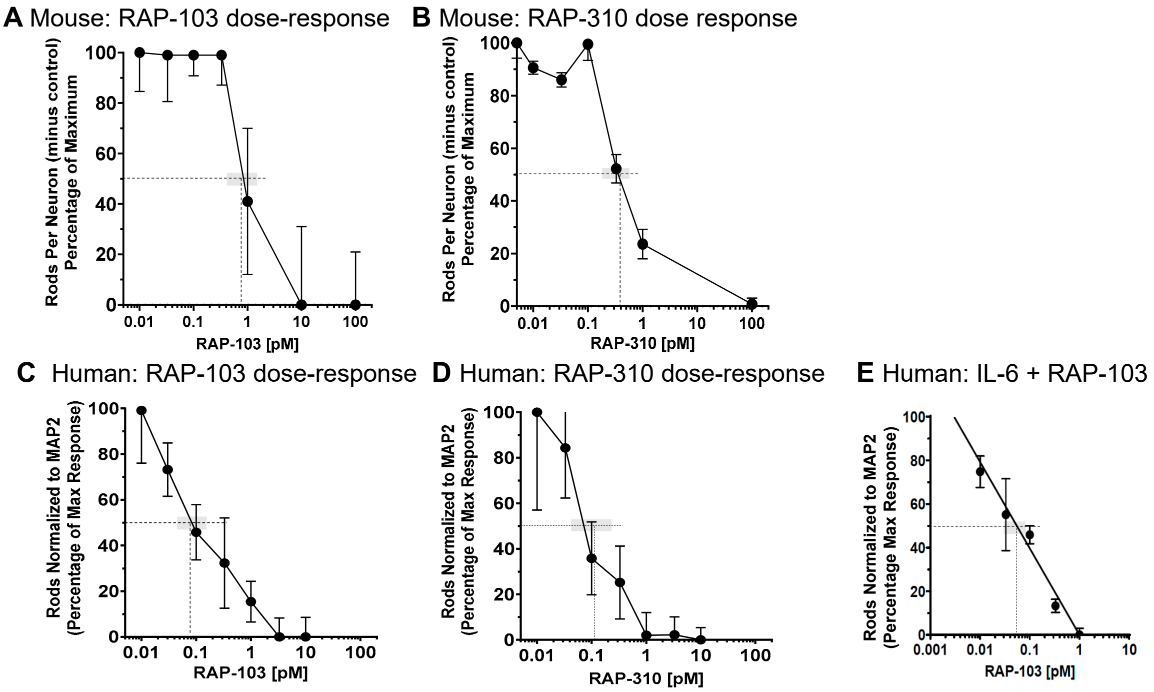

Figure S3. Log dose-response for inhibition by RAP-103 and RAP-310 of cofilactin rod formation induced by Aβd/t in mouse and human neurons and by IL-6 in human neurons. Data from Figure 4 and Figure 5C are replotted with a log dose scale for the RAPs after subtracting the control rod-response from the rod responses with different concentrations of RAP and then normalizing the rod values to the highest response set as 100%. The range of the likely EC_{50} value calculated from the S.D. of points on each plot is shown by the gray bar across the horizontal dashed line at 50%.

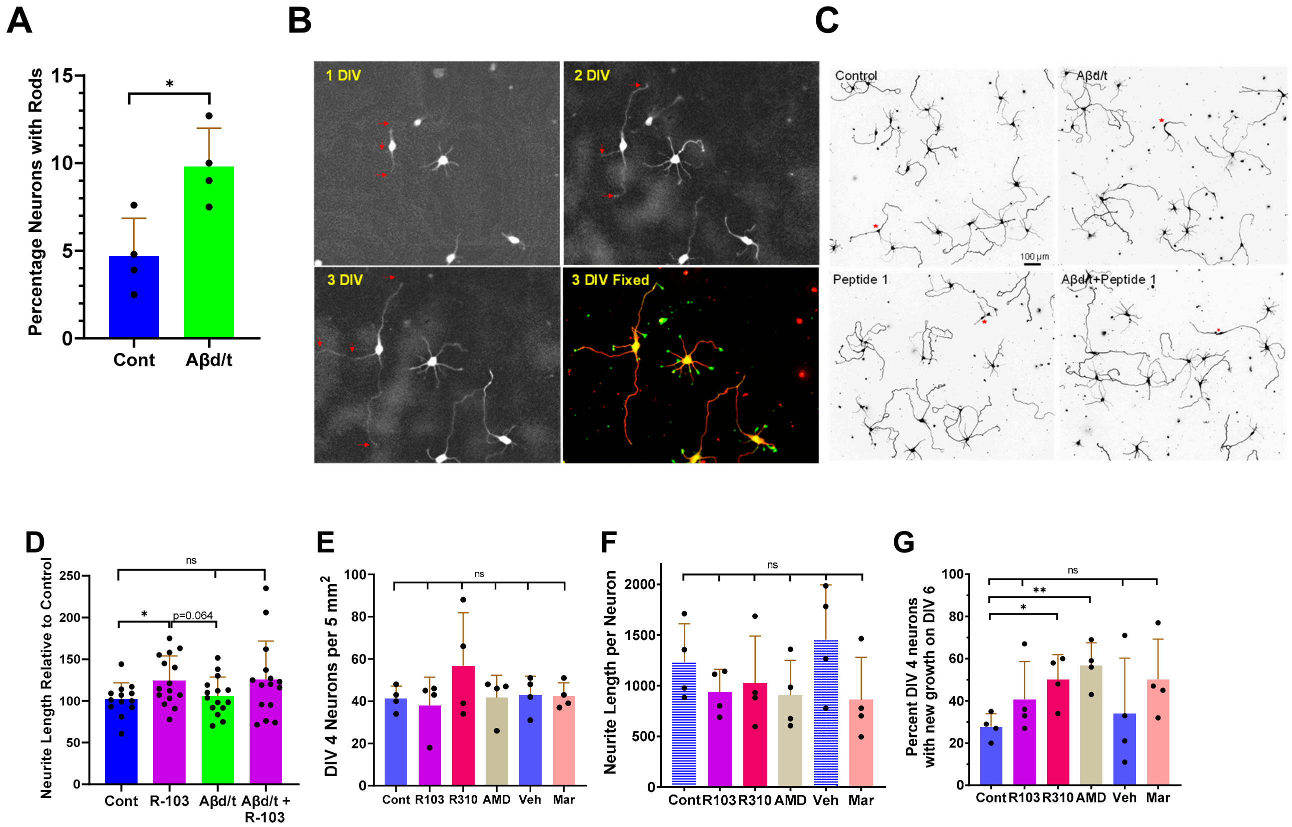

Figure S4. Neurite outgrowth and morphology in rodent neurons exposed to Aβd/t ± RAPs or CCR antagonists at sub-nM to μM concentrations. (**A**) E18 rat hippocampal neurons treated on DIV 1 with Aβd/t and fixed on DIV 3 show an Aβd/t-induced rod response although it is only about half of what is observed in older (6 DIV) cultures (e.g. see **Figure 2A,B** and **Figure 4A,B**). (**B, C**) Neurons were loaded for 2 h with the fluorescent vital dye, NeuO, about 2 h after plating. The neurons were imaged on DIV 1, reloaded for 2 h with NeuO and were untreated or treated with Aβd/t ± RAP-103 (Peptide 1) and imaged again on DIV 2 and 3 after which they were fixed and immunolabeled for NF-H (red) and cofilin (green) and then imaged again. (**B**) Region of a control dish showing the same neurons on DIV 1, 2 and 3 after NeuO loading and after fixation and immunolabeling. Bright green cofilin-immunolabeled growth cones are similar across all treatments. (**C**) Inverted b/w images of immunolabeled cultures from each of the different treatments (fixed on DIV 4). Pyramidal cells constitute ~95% of neurons with ~5% bipolar neurons present regardless of treatment. The number of primary neurites per cell (5.5 ± 0.3 S.D.) remained constant between treatments (n=3). (**D**) Total length of neurites/neuron treated with Aβd/t ± RAP-103 or RAP-103 alone were compared relative to the untreated controls set as 100. RAP-103 treatment showed a small but significant increase in outgrowth length but there were no other significant changes with Aβd/t ± RAP103 (n=4). **(E)** E16.5 mouse neurons were evaluated for survival when untreated or treated with 1 μM RAP peptides or CCR antagonists (n=2). None of the treatments were significantly different in survival compared to untreated cultures although RAP310 showed some high variability. The 1 μM concentration used is 10^6-fold higher than the EC_{50} for rod inhibition by RAPs 103 and 310 but only 10^3-fold higher than the EC_{50} for AMD3100 and Maraviroc. **(F)** Total neurite length per neuron for cells treated identically as in **(E)** showed no significant differences between treatments when fixed and measured on DIV 6 (n=2). (**G**) Neurite outgrowth between DIV 4 and 6 in culture was quantified from images captured using oblique illumination (n=2). Neurons treated with both RAP-310 and AMD3100 had a significantly higher percentage of neurons with neurites that elongated between days 4 and 6, although the limited number of

observations and the finding that were no significant differences in total neurite length per neuron between treatments (**F**), suggests that these differences are not biologically relevant.
